# Magneto-Structural Coupling in the Antiferromagnetic Copper Orthoniobate Cu_3_Nb_2_O_8_

**DOI:** 10.1021/acsomega.6c02538

**Published:** 2026-06-23

**Authors:** Diego da Silva Evaristo, Romualdo Santos Silva, Raí Figueredo Jucá, Luiz Fernando Lobato da Silva, Gilberto Dantas Saraiva, Waldeci Paraguassu Feio, Javier Gainza, João Elias Rodrigues, Jose Luis Martínez, Jose Antonio Alonso, Nilson dos Santos Ferreira, Marcelo Andrade Macêdo, Antonio Joel Ramiro de Castro

**Affiliations:** † Faculty of Education Sciences and Letters of Sertão Central, State University of Ceará, 63902-098 Quixadá, Brazil; ‡ Instituto de Ciencia de Materiales de Madrid (ICMM), CSIC, E-28049 Madrid, Spain; § Department of Physics, 74391 Federal University of Sergipe, 49100-000 São Cristóvão, Sergipe, Brazil; ∥ Institute of Criminalistics, Scientific Police of Pará, 68507-000 Marabá, PA, Brazil; ⊥ Institute of Exact and Natural Sciences, Federal University of Pará, 66075-110 Belém do Pará, Brazil; # European Synchrotron Radiation Facility (ESRF), 71 Avenue des Martyrs, 38000 Grenoble, France; ∇ University Federal of Ceará, 63902-580 Quixadá, CE, Brazil

## Abstract

The structural, vibrational, electronic, optical, and magnetic properties of triclinic Cu_3_Nb_2_O_8_ were investigated through a combined experimental and theoretical approach. Synchrotron X-ray diffraction confirmed the centrosymmetric *P*1̅ structure, while Raman and infrared spectroscopy enabled the identification of the vibrational modes, supported by lattice-dynamics calculations. Density functional theory calculations indicated that the electronic states near the conduction band minimum are mainly associated with Cu 3d orbitals, whereas the valence band is predominantly composed of O 2p states. Magnetic susceptibility measurements revealed two antiferromagnetic transitions at *T*_1_ = 26.09 K and *T*_2_ = 24.15 K. Temperature-dependent Raman analyses evidenced anomalous phonon behavior between 20 and 60 K, indicating spin-phonon interactions associated with the magnetic ordering process. In addition, magnetocaloric measurements exhibited a magnetic entropy variation centered near the Néel temperature, reinforcing the presence of magnetoelastic coupling in Cu_3_Nb_2_O_8_. These results provide a comprehensive description of the interplay between lattice dynamics, electronic structure, and magnetic ordering in this copper orthoniobate system.

## Graphical abstract

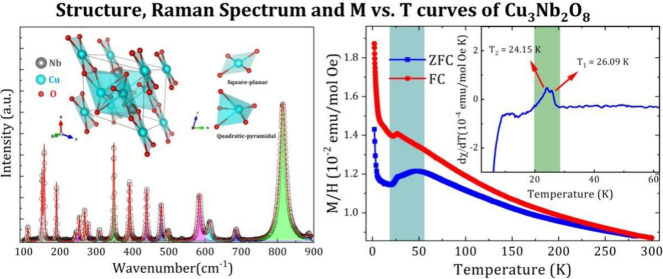

## 1 Introduction

The complex transition metal oxides such as copper niobium oxygen systems have garnered significant interest because of their rich interplay between structural distortions, electronic correlations, and magnetic interactions.^1–5^ Such complex systems can be obtained through compositional adjustment and are commonly divided into two classes depending on whether copper exhibits a +1 or +2 oxidation state.^6^ Additionally, these materials, in which spontaneous ferroelectricity and magnetic ordering coexist and are mutually coupled, have emerged as one of the most fascinating research topics of the past decade.^2^ The coupling between ferromagnetic and ferroelectric order parameters at operating temperatures may enable the development of novel magnetoelectric devices that can be independently manipulated by electric and magnetic fields.^5^ In particular, magnetoelectric coupling, have gained significant attention because of their fascinating physical properties and broad technological potential.^2^ However, magnetism requires partially occupied orbitals, while ferroelectricity tends to require fully occupied orbitals, making this coupling intrinsically difficult to achieve. This therefore necessitates the exploration of new mechanisms, such as that exploiting crystal symmetry.^5^

Recently, materials such as RbFe­(MoO_4_)_2_,^7,8^ CaMn_7_O_12_,^9–11^ MnSb_2_O_6_,^12,13^ and Cu_3_Nb_2_O_8_ ^3,5,14,15^ were discovered as a new family of multiferroic exhibiting the coexistence of structure and magnetic chiralities.^2^ Cu_3_Nb_2_O_8_, in particular, is classified as a p-type semiconductor oxide with a band gap of 1.21 eV,^16^ which crystallizes in a centrosymmetric triclinic symmetry belonging to the *P*1̅ space group.^3,15,17^ Its magnetic properties are characterized by complex behavior and magnetic phase transitions at low temperatures. As the temperature decreases, Cu_3_Nb_2_O_8_ undergoes two magnetic phase transitions. The first transition at *T*_1_ ≈ 26.5 K marks the beginning of antiferromagnetic ordering (AFM) and the emergence of ferroelectric properties (magnetoelectric coupling). Then, at *T*_2_ ≈ 24.2 K, a second transition occurs that alters the spin configuration; that is, the spins assume a coplanar helical configuration. This spin rotation is responsible for breaking the spatial inversion symmetry (*P*1̅ → *P*1) and generating electrical polarization (multiferroicity).^2,5,14^ Although the transition from *P*1̅ to *P*1 formally relaxes the Raman selection rules and could allow additional modes, no new Raman bands are observed in the present study. This suggests that the inversion symmetry breaking is associated with very small lattice distortions, likely driven by magnetic ordering. Further experimental and theoretical investigations are required to fully clarify the nature of this transition and its impact on lattice dynamics. The magnetic properties associated with Cu_3_Nb_2_O_8_ make it a strong candidate for membership in a new class of multiferroics, where the polarization is perpendicular to the spin rotation plane.^14,15^ Beyond its potential applications, its dielectric properties are being explored in low-temperature cofired ceramics (LTCC)^18^ and photocatalysis.^16^ It also shows favorable optical properties,^6^ and is reported as a strong candidate to present good thermoelectric performance,^19^ featuring a relatively low thermal conductivity of 6.1 W/m·K,^20^ which is a key requirement for potential thermoelectric applications.

The structural, electrical, and magnetic properties of Cu_3_Nb_2_O_8_ have been widely discussed in literature. However, to date, little is known about its vibrational properties. In this work, we comprehensively characterize the triclinic *P*1̅ phase of Cu_3_Nb_2_O_8_. First, the electronic band structure and the corresponding density of states (DOS) are investigated through first-principles calculations. Second, we study the nature of the experimentally observed vibrational modes employing the same theoretical framework to determine the vibrational wavenumbers, atomic vibration patterns, and symmetry characteristics associated with each Raman-active phonon mode.

Furthermore, this study explores the vibrational properties at room temperature and the Magnetoelastic couplings at low temperatures, as revealed by Raman spectroscopy. Finally, the magnetic phase transitions are thoroughly elucidated in this report.

## 2 Experimental and Computational Methodologies

### 2.1 Copper Orthoniobate Cu_3_Nb_2_O_8_

The Cu_3_Nb_2_O_8_ sample was synthesized through a conventional solid-state reaction route. The procedure involved the precise measurement of the starting reagents: copper oxide (CuO, Sigma-Aldrich) and niobium pentoxide (Nb_2_O_5_, Sigma-Aldrich), ensuring stoichiometric quantities. The mixture was initially homogenized and subsequently heat-treated at 950 °C for 12 h, all conducted under ambient atmospheric conditions.

### 2.2 Characterization Techniques

Synchrotron X-ray diffraction (SXRD) measurement of powder was performed at the ID22 beamline of the European Synchrotron Radiation Facility (ESRF),^21^ located in Grenoble. Diffraction data were acquired in continuous scanning mode in the 2θ range from 1° to 40° with a wavelength of λ = 0.35418 Å (35 keV). The sample was sealed in a 0.5 mm diameter glass capillary and measured under continuous rotation. The resulting SXRD pattern was subsequently analyzed by Rietveld refinement using the GSAS-II program.^22^ Fourier-transform infrared (FT-IR) spectra were acquired using a Bruker infrared spectrometer in the wavenumber range of 200–1250 cm^–1^. Raman experiments under low-temperature conditions were carried out using a THMS 600 cryogenic configuration. The spectra were recorded on a Jobin-Yvon T64000 triple-monochromator operating in subtractive mode, providing a spectral resolution of 2 cm^–1^. Excitation was achieved using the 514.5 nm emission line of an Ar^+^ laser, while signal collection was performed through an Olympus microscope objective with a numerical aperture of 0.35. Magnetic measurements were performed using a SQUID MPMS3 magnetometer (Quantum Design). Measurements were taken in the temperature range of 2 to 300 K under magnetic fields up to 7 T. Electron paramagnetic resonance (EPR) measurements were performed using a Bruker EMXmicro spectrometer. The spectra were acquired in the X-band region using a microwave frequency of 9.5 GHz. The magnetic field was scanned up to *H* = 700 mT, and the resulting X-band spectra were integrated to determine the *g*-factor.

### 2.3 Theoretical Calculations

The electronic and optical characteristics of CNO investigated by means Density Functional Theory (DFT) calculations using the Cambridge Serial Total Energy Package (CASTEP).^23^ The electronic structure was described using norm-conserving pseudopotentials,^24^ while exchange-correlation interactions were accounted for within the Generalized Gradient Approximation (GGA) framework through the Perdew–Burke–Ernzerhof (PBE) functional.^25^ Brillouin Zone (BZ) integrations were performed employing a 3 × 3 × 2 Monkhorst–Pack *k*-point sampling scheme.^26^ A plane-wave basis set with a high energy cutoff of 800 eV was applied to ensure accurate convergence. To address the strong on-site Coulomb interactions of the localized d electrons, particularly for Cu atoms, the DFT+*U* method A plane-wave basis set with an energy cutoff of 800 eV was adopted to guarantee well-converged results. Given the strongly correlated nature of localized d electrons, especially in Cu atoms, the DFT+*U* approach^27,28^ was employed, incorporating the Hubbard *U* parameter into the atomic orbitals to properly account for on-site Coulomb interactions. Structural relaxation was carried out via the Broyden–Fletcher–Goldfarb–Shanno (BFGS) algorithm^29^ until the following convergence thresholds were simultaneously met: maximum energy change of 1.0 × 10^–6^ eV/atom, maximum ionic force of 0.03 eV/Å, maximum stress of 0.1 GPa, and maximum atomic displacement of 0.001 Å. Following geometry optimization, the electronic band structure and related quantities were obtained by evaluating the electronic wave functions along high-symmetry points of the BZ, according to the *k*-path: Γ­(0.000, 0.000, 0.000) – F­(0.000, 0.500, 0.000) – Q­(0.000, 0.500, 0.500) – Z­(0.000, 0.000, 0.500) – Γ­(0.000, 0.000, 0.000). All calculations were carried out considering a triclinic unit cell (space group *P*1̅) containing a total of 13 ions.

The intermolecular interactions present in the CNO structure were investigated by Hirshfeld surface calculations using Crystal Explorer 17 package, based on crystallographic data obtained from the Inorganic Crystal Structure Database (ICSD, entry no. 330476).^30^ The surfaces were generated from the normalized contact function (*d*_norm_), which incorporates the distances to the nearest atoms outside (*d*_e_), and inside (*d*_i_), the surface, as well as their van der Waals radii (*r*_vdW_).^23^ Furthermore, two-dimensional (2D) fingerprint diagrams were employed to analyze and quantify the intermolecular contact contributions associated with the crystal arrangement. The presence of structural voids and empty regions within the primitive unit cell was also examined from electron-density isosurfaces calculated with an isovalue of 0.01 atomic units (a. u.).^24^

## 3 Results and Discussion

### 3.1 Structural and Hirshfeld Surface Analysis

The crystal structure was determined by SXRD analysis at room temperature (300 K). Figure 1a presents the SXRD powder diffraction pattern of CNO, together with the simulated pattern obtained from the Rietveld refinement. The Rietveld refinement results confirm that the sample crystallizes in the triclinic structure with space group *P*1̅ (No. 2), in agreement with the ICDD database (entry No. 00-033-0476).^30^ A secondary phase corresponding to approximately 13% of the sample was identified as CuO with monoclinic symmetry and space group *C*_2/*c*_ (no. 9). The structural parameters obtained from the refinement, including atomic positions, occupancies, bond lengths, bond angles, and refinement quality factors, are listed in Table 1. Conventional laboratory XRD measurements using Cu Kα radiation, followed by Rietveld refinement (Figure S1 and Table S1 of the Supporting Information), corroborate the same CNO crystal structure determined from the SXRD analysis. The crystal structure contains two crystallographically nonequivalent Cu^2+^ sites: Cu1, located at the Wyckoff position 1a (inversion center) with square-planar coordination, and Cu2, located at the Wyckoff position 2i (general position) with square-pyramidal coordination. These Cu atoms form sawtooth chains composed of edge-sharing CuO_4_ square-planar units extending along the *a* axis and generating Cu–O layers within the *ac* plane. The layers are separated along the *b* axis by nonmagnetic Nb atoms, as illustrated in Figure 1b. The oxygen atoms O1, O2, O3, and O4 are associated with the coordination environment and bond angles involving Cu1 and Cu2, as summarized in Table 1.

**Figure 1:**
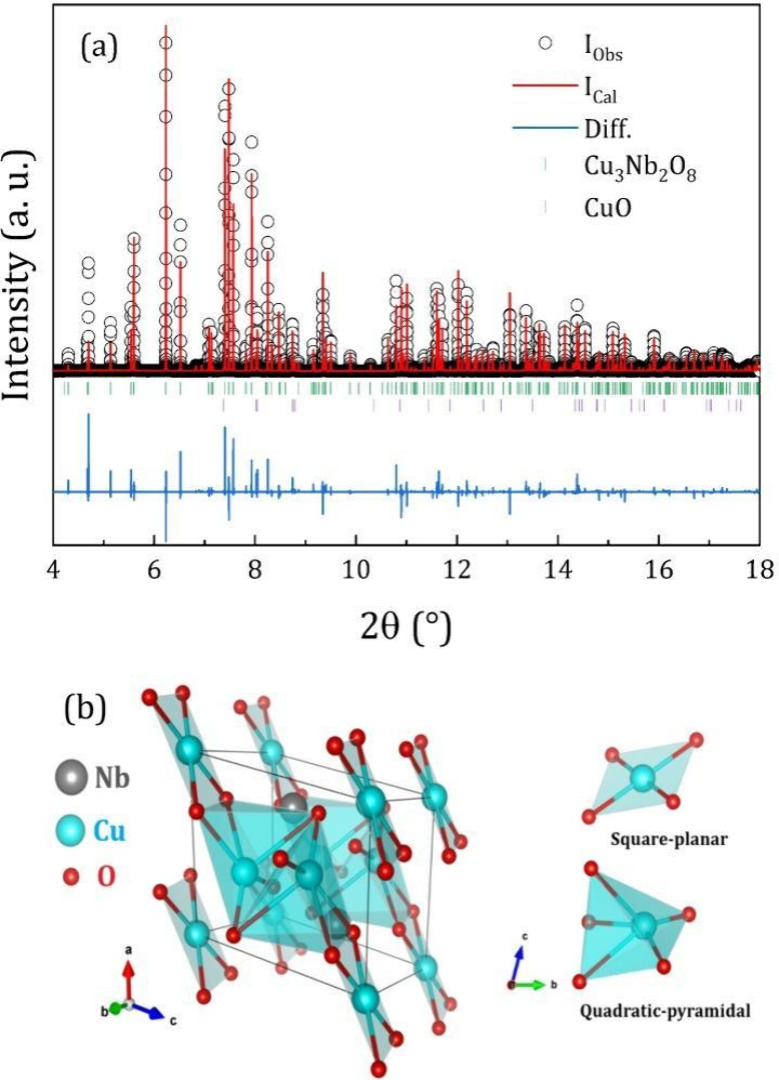
(a) Rietveld refinement of the SXRD pattern for CNO. The observed and calculated diffraction profiles are represented by white circles and solid red lines. (b) The triclinic phase unit cell of CNO (space group *P*1̅ (No. 2)) forms sawtooth Cu–O chains along the *a*-axis and Cu–O layers in the *ac*-plane, which are separated by nonmagnetic Nb atoms along the *b*-axis.

**Table 1:** Rietveld Refinement Data, Atomic Positions, Lattice Parameters, Quality Factors, Bond Angles and Lengths

atom	Pos. Wyck.	*x*	*y*	*y*	Occup.	*B*
**Cu1**	1a	0.00000(0)	0.00000(0)	0.00000(0)	0.97(1)	0.011(3)
**Cu2**	2i	0.45948(0)	0.07446(5)	0.23480(7)	0.96(7)	0.011(7)
**Nb**	2i	0.22150(1)	0.54189(5)	0.65185(9)	0.98(9)	0.009(1)
**O1**	2i	0.23282(2)	0.20455(6)	0.90199(8)	0.90(1)	0.012(3)
**O2**	2i	0.26063(1)	0.74180(9)	0.82329(5)	1.00(0)	0.037(0)
**O3**	2i	0.35887(5)	0.73744(0)	0.36616(7)	0.92(8)	0.014(4)
**O4**	2i	0.17800(5)	0.30236(2)	0.40224(1)	0.94(2)	0.017(0)
lattice parameters:						
*a* = 5.18258(5) (Å)		*b* = 5.48305(1) (Å)		*c* = 6.01200(8) (Å)		*V* = 148.49(1) (Å^3^)
α = 72.529(1) (°)		β = 83.417(5) (°)		γ = 65.681(6) (°)		ρ = 5.52(0) (g/cm^3^)
**quality factors**: RF = 3.53%, W_R_ = 16.15%						
bond length (Å) and angle [°]:						
**Cu(1)–O(1)**	**Cu(1)–O(2)**	**Cu(2)–O(1)**	**Cu(2)–O(1)**	**Cu(2)–O(2)**	**Cu(2)–O(3)**	**Cu(2)–O(4)**
1.657(3)	2.012(3)	1.901(9)	2.124(5)	2.011(6)	2.062(0)	1.942(8)
**Cu(1)–O(1)–Cu(2)**	111.50(3)	**Cu(1)–O(2)–Cu(2)**	90.33(0)			

To complement the structural data related to chemical bonding in the CNO system, a theoretical analysis based on Hirshfeld surface mapping was carried out using the unit cell of the crystal structure (see Figure 2a). The insets in Figure 2b–f display the corresponding Hirshfeld surfaces mapped with the normalized contact distance (*d*_norm_) function. The surface is represented by a color gradient that reflects the intensity of intermolecular interactions: red indicates contacts at distances shorter than the van der Waals (*r*_vdW_) radius; white corresponds to distances close to the *r*_vdW_ radius, and blue represents contacts occurring at distances greater than the *r*_vdW_ radius.^25,26^ The reddish regions predominantly found around oxygen atoms, indicate stronger interatomic interactions, suggesting either covalent bonds or relevant noncovalent interactions.

**Figure 2:**
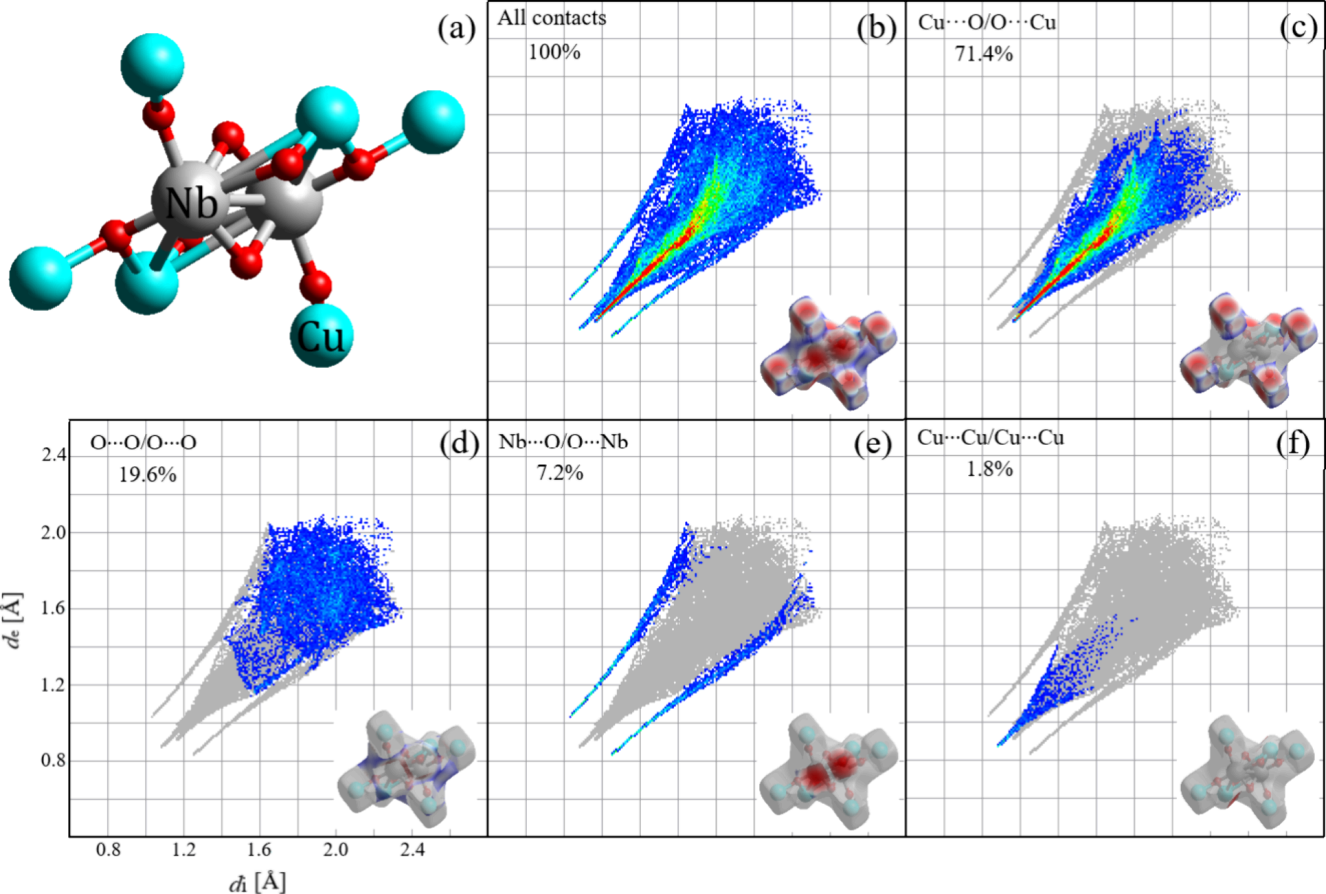
(a) Molecular structure of the CNO crystal; (b) 2D fingerprint plots of the CNO crystal: (b) all contact, (c) Cu···O/O···Cu, (d) O···O, (e) Nb···O/O···Nb and (f) Cu···Cu contacts.

Figure 2b–f present a quantitative analysis of the intermolecular interactions through 2D fingerprint mappings. Figure 2b shows the cumulative pattern, which gathers all the chemical interactions present in the CNO crystal lattice. Figure 2c corresponds to the Cu···O/O···Cu ionic interaction, with a significant contribution of 71.4%. This interaction strengthens the structure of the compound due to the strong electrostatic attraction between Cu^2+^ and the oxygen anions (O^2–^). Moreover, Figure 2d shows the O···O interaction, of van der Waals nature, which contributes 19.6%. Meanwhile, Figure 2e represents the Nb···O/O···Nb ionic interaction, with a much smaller contribution of only 7.2%, suggesting a stable but less dominant ionic environment around the niobium atoms. Figure 2f shows the Cu···Cu interaction, with the smallest observed contribution of only 1.8%. This interaction, possibly of a metallic or van der Waals nature, may be related to weak exchange magnetic couplings between the Cu^2+^ ions.^4^ The results confirm that the stability of CNO is primarily due to the electrostatic interactions that are typical of polar metal oxides.

### 3.2 Electronic Structure and PDOS

The Band Structure and Projected Partial Density of States (PDOS) were computed based on the experimental crystallographic data obtained for CNO (see Table 1). Examining the band structure is fundamental to understanding the electronic properties of materials, as it yields critical information regarding energy levels and the distribution of electronic states within the periodic lattice, thereby elucidating characteristics such as electrical conductivity, magnetic ordering, and optical response. In the DFT+*U* calculations, a Hubbard *U* parameter of 6.0 eV was assigned to the Cu 3d orbital to properly describe on-site Coulomb interactions, following the approach reported by Moore et al.^31^

The band structure results, shown in Figure 3 a,b, reveal a band gap of 1.37 eV in the minority spin channel, spanning from the Z point to the F point, which is consistent with semiconducting behavior. This theoretically predicted band gap value is in good agreement with the experimentally reported value of *E*_g_ = 1.21 eV for the CNO.^13^ As evidenced by the PDOS analysis presented in Figure S2, this gap is primarily governed by the localized Cu 3d orbitals (Figure 3c). The PDOS decomposition resolves the total density of states into individual atomic and orbital contributions, thereby clarifying the electronic structure and bonding characteristics of the material. In this context, Figures 3c and S2a highlights a substantial contribution of the Cu 3d orbitals to the states at the Conduction Band Minimum (CBM) in the vicinity of the band gap, underscoring their central role in determining the observed semiconducting behavior. Furthermore, the PDOS analysis indicates that the Nb 4d orbitals contribute significantly to the electronic states in the conduction band above approximately 3 eV. In contrast, the valence band is predominantly composed of O 2p states, with comparatively smaller yet non-negligible contributions from Cu 3d orbitals in the vicinity of the Fermi level. To prevent potential misinterpretation, it is worth emphasizing that the Cu 3d states are not confined solely to the CBM region, but rather extend over a broad energy range around the Fermi level. The inclusion of the on-site Coulomb interaction term (U) induces a splitting of the Cu 3d manifold into lower-energy occupied states and higher-energy unoccupied states, reflecting the strong electronic correlations inherent to the system. Each Cu^2+^ ion adopts a 3d^9^ electronic configuration, corresponding to nine localized d-electrons and an effective spin of *S* = 1/2. These localized magnetic moments constitute the primary source of magnetism in the compound. The magnetic behavior arises from the coupling between neighboring Cu spins via superexchange interactions mediated by bridging oxygen atoms. Consequently, the Cu 3d orbitals play a dual role, they contribute to both the occupied valence states and the unoccupied states near the CBM, while simultaneously governing the magnetic properties of the system.

**Figure 3:**
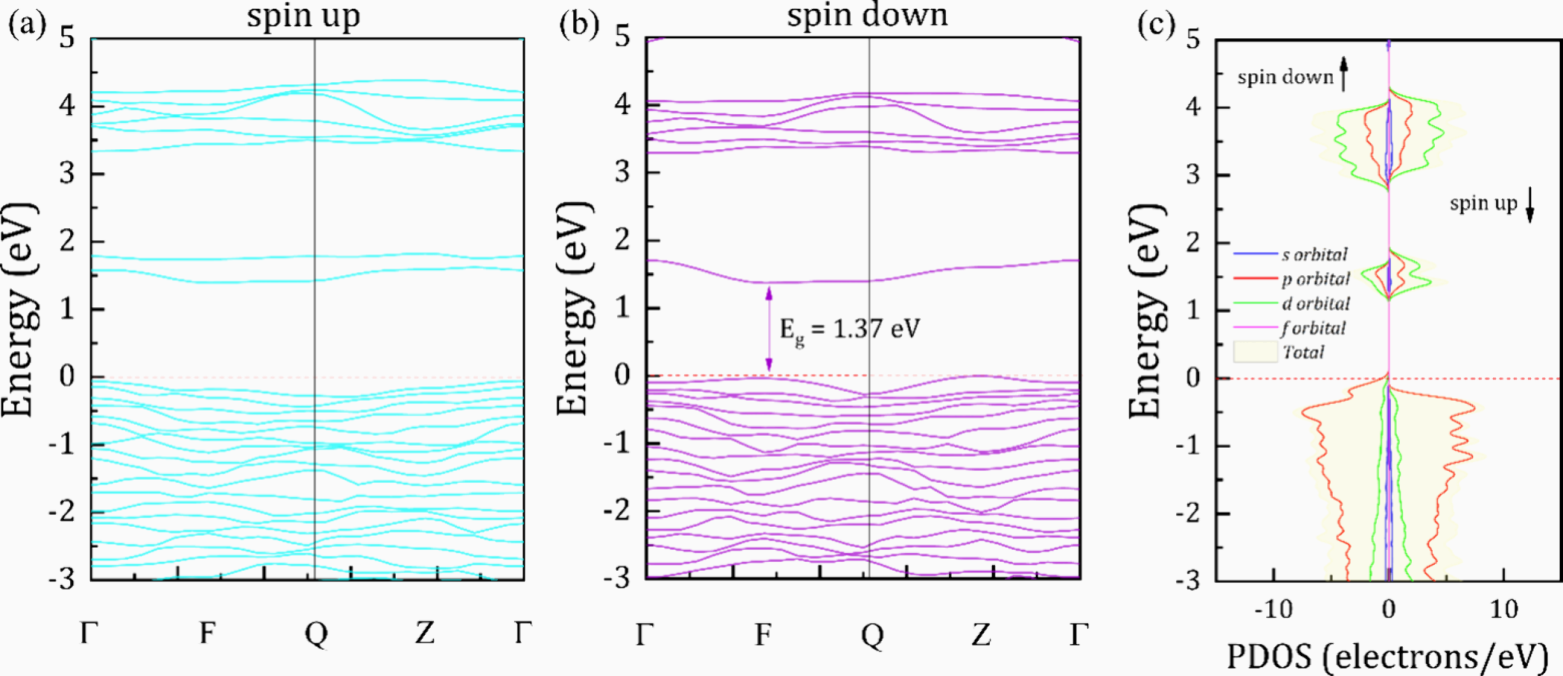
(a,b) Electronic band structure as a function of energy for both spin-up and down channels calculated via DFT, respectively. (c) Orbital contributions through projected density of states (PDOS) for the CNO crystal.

The spin-resolved PDOS for up- and down-spin electrons (Figure 3c) reveals an asymmetric distribution across all atomic species, indicative of a net magnetic character. This asymmetry is most pronounced in the Cu 3d orbitals. Additionally, the polarized optical response depicted in Figure S3 shows distinct variations in optical absorption along the 001, 010, and 100 crystallographic directions, confirming that CNO exhibits anisotropic optical behavior.

### 3.3 Vibrational Properties under Ambient Conditions

According to the analysis based on the irreducible representations of the factor group *C*_*i*_, the crystal symmetry of CNO indicates that the 3N degrees of freedom associated with the coupled vibrations of the 13 atoms in the primitive unit cell generate 39 internal vibrational modes, given by Γ_total_ = 18Ag + 21Au. Among these 39 modes, 36 correspond to optical modes at *k* = 0, resulting in Γ_optic_ = 18Ag + 18Au, while the remaining three correspond to acoustic modes, expressed as Γ_acoustic_ = 3Au.^32^ Figure 4 a,b present the deconvoluted experimental Raman and infrared (IR) spectra of CNO at room temperature. Based on the lattice dynamics (LD) calculations, the vibrational wavenumbers of the experimentally observed and theoretically calculated Raman- and IR-active modes, together with their corresponding assignments, are summarized in Table 2.

**Table 2:** Analysis of Vibration Modes for the Cu_3_Nb_2_O_8_: Experimental Raman Modes (ω_R_), Experimental IR Modes (ω_IR_), Calculated Wavenumbers (ω_cal_), Irreducible Representations (Irrep.), and Their Assignments

ω_R_ (cm^–1^)	ω_cal_ (cm^–1^)	Ired. Repr.	assignments^†^	ω_IR_ (cm^–1^)	ω_cal_ (cm^–1^)	Ired. Repr.	assignments^†^
886	886	A_g_	ν_s_ [CuO_5_] + δ [CuO_4_], motion of Cu in the CuO_5_ plus motion of Nb atoms	853	912	A_u_	strech of O–Cu–O in the [CuO_5_] + δ [CuO_4_], motion of Cu in the CuO_5_ plus motion of Nb atoms
815	883	A_g_	ν_s_ [CuO_5_] + δ [CuO_4_], motion of Cu in the CuO_5_ plus motion of Nb atoms	814	813	A_u_	strech of O–Cu–O in the [CuO_5_] + ν [CuO_4_], motion of Cu in the CuO_5_ plus motion of Nb atoms
685	734	A_g_	ν_as_ [CuO_5_] + δ [CuO_4_], motion of Cu in the CuO_5_ plus motion of Nb atoms	724	740	A_u_	ν_as_ [CuO_4_] + bend [CuO_5_], motion of Cu in the CuO_5_ plus motion of Nb atoms
612	679	A_g_	δ [CuO_5_ + CuO_4_], motion of Cu in the CuO_5_ plus motion of Nb atoms	669	672	A_u_	strech of O–Cu–O in the [CuO_5_] + δ [CuO_4_], motion of Cu in the CuO_5_ plus motion of Nb atoms
584	656	A_g_	δ [CuO_5_ + CuO_4_], motion of Cu in the CuO_5_ plus motion of Nb atoms	631	657	A_u_	strech of O–Cu–O in the [CuO_5_] + ν_as_ [CuO_4_], motion of Cu in the CuO_5_ plus motion of Nb atoms
536	607	A_g_	δ [CuO_5_], bend and a stretch in different O–Cu–O bond of the CuO_4_, motion of Cu in the CuO_5_ plus motion of Nb atoms	598	594	A_u_	δ [CuO_5_ + CuO_4_], motion of Cu in the CuO_5_ plus motion of Nb atoms
497	562	A_g_	δ [CuO_5_ + CuO_4_], motion of Cu in the CuO_5_ plus motion of Nb atoms	499	551	A_u_	δ [CuO_5_ + CuO_4_], motion of Cu in the CuO_5_ plus motion of Nb atoms
479	540	A_g_	δ [CuO_5_], bend and a stretch in different O–Cu–O bond of the CuO_4_, motion of Cu in the CuO_5_ plus motion of Nb atoms	440	441	A_u_	δ [CuO_5_ + CuO_4_], motion of Cu in the CuO_5_ plus motion of Nb atoms
439	464	A_g_	δ [CuO_5_ + CuO_4_], motion of Cu in the CuO_5_ plus motion of Nb atoms	419	409	A_u_	δ [CuO_5_ + CuO_4_], motion of Cu in the CuO_5_ plus motion of Nb atoms
348	396	A_g_	δ [CuO_5_ + CuO_4_], motion of Cu in the CuO_5_ plus motion of Nb atoms	390	381	A_u_	δ [CuO_5_ + CuO_4_], motion of Cu in the CuO_5_ plus motion of Nb atoms
309	389	A_g_	δ [CuO_5_ + CuO_4_], motion of Cu in the CuO_5_ plus motion of Nb atoms		299	A_u_	strech of O–Cu–O in the [CuO_5_] + δ [CuO_4_], motion of Cu in the CuO_5_ plus motion of Nb atoms
277	330	A_g_	δ [CuO_5_ + CuO_4_], motion of Cu in the CuO_5_ plus motion of Nb atoms		285	A_u_	strech of O–Cu–O in the [CuO_5_] + δ [CuO_4_], motion of Cu in the CuO_5_ plus motion of Nb atoms
267	273	A_g_	Lib [CuO_4_] + ν_as_ [CuO_5_], motion of Cu in the CuO_5_ plus motion of Nb atoms		217	A_u_	strech of O–Cu–O in the [CuO_5_] + δ [CuO_4_], motion of Cu in the CuO_5_ plus motion of Nb atoms
253	246	A_g_	δ [CuO_4_] + ν_as_ [CuO_5_], motion of Cu in the CuO_5_ plus motion of Nb atoms		196	A_u_	T [NbO_6_ + CuO_5_ + CuO_4_]
245	211	A_g_	δ [CuO_4_ + CuO_5_], strong motion of Cu in the CuO_5_ plus motion of Nb atoms		189	A_u_	T [NbO_6_ + CuO_5_ + CuO_4_]
156	138	A_g_	T [NbO_6_] + δ [CuO_5_ + CuO_4_], motion of Cu in the CuO_5_		167	A_u_	T [NbO_6_ + CuO_5_ + CuO_4_]
150	131	A_g_	T [NbO_6_ + CuO_5_ + CuO_4_]		148	A_u_	T [NbO_6_ + CuO_5_ + CuO_4_]
111	116	A_g_	T [NbO_6_ + CuO_5_ + CuO_4_]		128	A_u_	T [NbO_6_ + CuO_5_ + CuO_4_]

**Figure 4:**
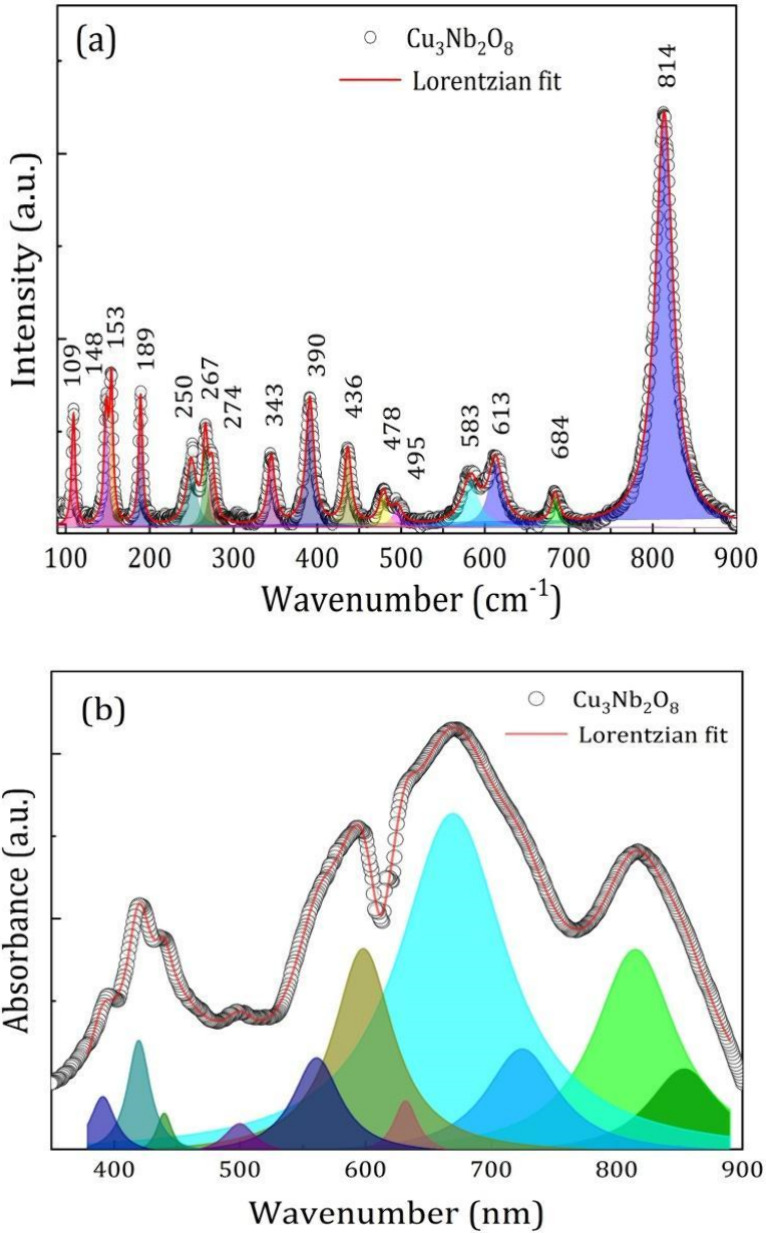
(a) Experimental Raman spectra of deconvoluted CNO in the range of 100–900 cm^–1^, and (b) experimental infrared spectra of deconvoluted CNO in the range of 350–900 cm^–1^.

To determine the assignment of the Raman and IR vibrational modes of CNO, LD calculations were employed to obtain the eigenvalues and atomic displacement patterns of the compound. This approach is based on a set of ionic potentials within a model that describes the material as a system of central units interacting through short-range classical potentials. Furthermore, this methodology has been successfully applied to the vibrational characterization of several materials.^27,28^ The force constants associated with these interactions were determined using the following relation:

$$f_{ij}=-\frac{1}{r}\frac{\partial U_{ij}\left(r\right)}{\partial r}$$ 1

where *f*_*ij*_ denotes the force constant, *U*_*ij*_ is the interaction potential between species *i* and *j*, and *r* is the distance separating the interacting species. For the Cu–Cu and O–O interactions we have used as starting parameters the *U*_*ij*_(*r*) Buckingham potential reported previously^33^ as the starting parameters.

In the interactions among Cu–O and Nb–O in the first neighborhood we have considered the partially covalent model, represented by a linear interatomic force of the form:

$$f_{ij}\left(r\right)=f_{2}-f_{3}r$$ 2

This linear force corresponds to the potential energy function *U*_*ij*_(*r*) of the following equation harmonic form:

$$U_{ij}\left(r\right)=f_{0}+f_{2}\frac{r^{2}}{2}$$ 3

where *f*_2_ and *f*_3_ are constants to be refined by a fitting of the experimental data.

The input parameters and the atomic positions applied to perform the lattice dynamics (LD) calculations for the CNO were taken from reference.^3^ The starting parameters used in the phonon calculations were performed with a Wilson’s FG matrix method and the VIBRATZ software package of developed by Dowty.^34^ Table 3 shows the final force constants used in LD (in mDyn/ Å), obtained after the refinement which was performed to obtain the best fit to the experimental data. These force constants refer to the B–O bond of the first neighborhood of each species (B = Cu, Nb, and O).

**Table 3:** Final Force Constants Used in LD (in mDyn/ Å), Obtained after the Refinement, Which Was Performed to Obtain the Best Fit to the Experimental Data

species	force constants (mDyn/Angs)
Cu–O	*f* = −4.7143*r* + 11.4429
Nb–O	*f* = −4.1429*r* + 10.1286
Cu–Cu	856.36 exp(−3.3333*r*) + 28.879*r* ^–3^
O–O	15919.33 exp (−4.5617*r*) – 15.379*r* ^–8^ – 4.6196*r* ^–3^

#### 3.3.1 A_g_ Raman Modes

All Raman-active modes exhibit the A_g_ irreducible representation. The high-wavenumber modes at 886 and 883 cm^–1^ are assigned to symmetric stretching vibrations of [CuO_5_] pyramidal units, coupled with bending deformations of [CuO_4_] polyhedra. The mode observed at 734 cm^–1^ corresponds to asymmetric stretching of [CuO_5_] combined with bending of [CuO_4_]. These three modes involve coordinated displacements of Cu atoms within CuO_5_ polyhedra and Nb atoms in the lattice. A series of midwavenumber modes located at 679, 656, 562, 540, 463, 396, 389, 330, and 211 cm^–1^ are attributed to bending vibrations of [CuO_5_ + CuO_4_], with contributions from Cu motion in CuO_5_ and Nb atomic displacements. The mode at 607 cm^–1^ represents a mixed vibration, involving bending of [CuO_5_], stretching of O–Cu–O bonds in [CuO_4_], and motion of both Cu (in CuO_5_) and Nb atoms. In the low-wavenumber region, the modes at 273 and 246 cm^–1^ are assigned, respectively, to librational plus coupled with asymmetric stretching of [CuO_5_] and bend of [CuO_4_] plus coupled with asymmetric stretching of [CuO_5_], both involving Cu (in CuO_5_) and Nb atomic motion. The translational (T) modes appear below 140 cm^–1^: the mode at 138 cm^–1^ arises from the translational motion of [NbO_6_] combined with bending of [CuO_5_ + CuO_4_], while the modes at 131 and 116 cm^–1^ are assigned to collective translations of [NbO_6_ + CuO_5_ + CuO_4_] units.

#### 3.3.2 A_u_ Infrared Modes

The infrared-active modes exhibit the A_u_ irreducible representation. The high- wavenumber modes observed at 913 and 672 cm^–1^ are assigned to coupled vibrations involving stretching of O–Cu–O bonds in [CuO_5_] polyhedra combined with bending deformations of [CuO_4_] units. These modes are characterized by significant displacements of Cu atoms within CuO_5_ and concomitant motion of Nb atoms in the lattice. The mode at 813 cm^–1^ arises from stretching vibrations of O–Cu–O in [CuO_5_] coordinated with stretching of [CuO_4_] bonds, while the mode at 657 cm^–1^ represents a combination of O–Cu–O stretching in [CuO_5_] and asymmetric stretching of [CuO_4_]. All these vibrational modes involve substantial participation of Cu atoms in CuO_5_ polyhedra and Nb atomic motion. A distinct mixed vibrational mode is observed at 740 cm^–1^, which is attributed to asymmetric stretching of [CuO_4_] coupled with bending of [CuO_5_], again involving motion of both Cu (in CuO_5_) and Nb atoms. In the midwavenumber range, the modes centered at 594, 551, 441, 409, and 381 cm^–1^ are all assigned to bending vibrations of [CuO_5_ + CuO_4_] complexes, with contributions from Cu motion in CuO_5_ and Nb atomic displacements. The lower- wavenumber modes at 299, 285, and 217 cm^–1^ maintain the pattern of O–Cu–O stretching in [CuO_5_] combined with [CuO_4_] bending, continuing to involve motion of both Cu (in CuO_5_) and Nb atoms. Finally, the low-wavenumber modes observed at 196, 189, 167, 148, and 128 cm^–1^ are assigned to translational motions (T) of the complete structural units [NbO_6_ + CuO_5_ + CuO_4_], representing collective vibrations of the entire lattice framework.

### 3.4 Magnetic Properties

The magnetization versus magnetic field (*M–H*) curves measured at temperatures of 1.8, 30, 100, and 300 K, shown in Figure 5a, indicate predominant antiferromagnetic (AFM) behavior, with the magnetization increasing as the temperature decreases. The low-field region, highlighted in the inset of Figure 5a, exhibits a slight variation in the slope of the curves. This feature is attributed to the contribution of CuO magnetic impurities present in the sample.^35^ Figure 5b presents the temperature dependence of the magnetic susceptibility measured under zero-field-cooled (ZFC) and field-cooled (FC) protocols for CNO. The obtained behavior is consistent with previous reports available in the literature for CNO.^2,5^

**Figure 5:**
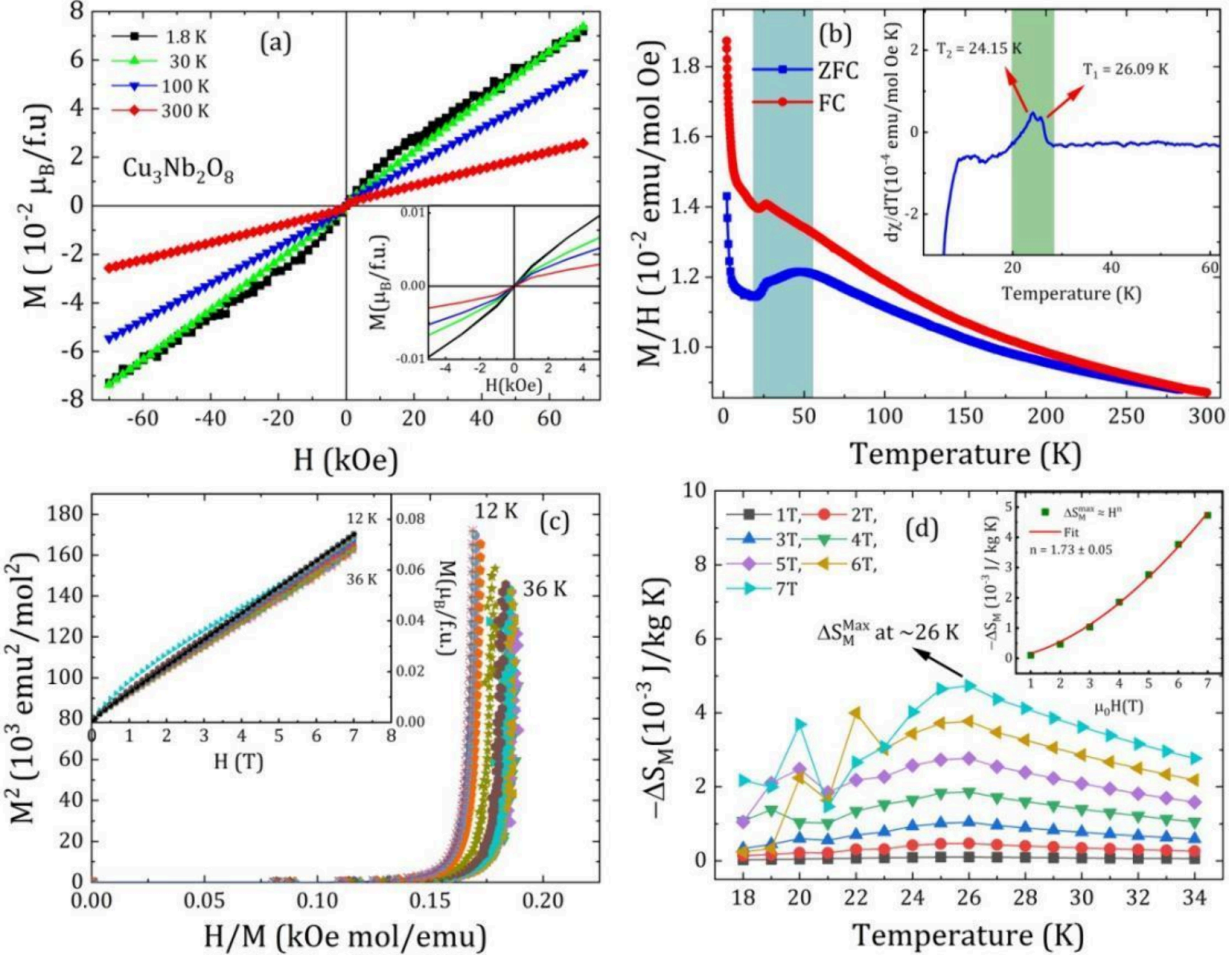
(a) Magnetization curves as a function of field (*M*–*H*) measured at different temperatures. (b) Magnetization curves as a function of temperature (*M*–*T*) for FC and ZFC modes, measured in a field of 100 Oe. The inset shows the derivative from susceptibility as a function of temperature. (c) Arrott plots (*H*/*M*–*M* ^2^). The inset shows isothermal *M*–*H* field-dependent up to 7 T measured from *T* = 12 to 36 K for the CNO sample (d) Temperature-dependent Δ*S*_M_ at various magnetic fields changes from 1 up to 7 T and.

The ZFC curve exhibits a gradual increase in magnetization with decreasing temperature down to approximately 60 K (blue curve), whereas the FC curve shows a continuous increase in magnetization down to approximately 30 K (red curve). However, within the temperature interval between 30 and 20 K, both the curves exhibit a decreasing magnetization tail, which is characteristic of low-temperature antiferromagnetic (AFM) ordering, as previously reported for CNO.^8–10^ Specifically, the decrease in magnetization observed in the ZFC curve starts at approximately 60 K, coinciding with the deviation from the Curie–Weiss behavior in the magnetic susceptibility (Figure S4). This temperature is close to the magnetic ordering temperature, indicating the onset of possible exchange interactions from this temperature onward.^2,5^ Below ∼20 K, a significant increase in both FC and ZFC curves is observed as the temperature decreases, which can be associated with the contribution of CuO magnetic impurities present in the sample.^35^ Crucially, the susceptibility derivative (dχ/d*T*) presents two peaks at *T*_1_ = 26.1 K and *T*_2_ = 24.2 K (see Figure 5b), indicating two successive magnetic transitions present in CNO. This result aligns with previous experimental studies on both polycrystalline^3–5^ and monocrystalline^2^ samples. The first observed transition (*T*_1_) corresponds to the onset of incommensurate magnetic ordering in the *T*_N_ = 26.1 K.^5^ Subsequently, the system develops a helical magnetic structure coupled to electric polarization, breaking crystallographic inversion symmetry (from *P*1̅ to *P*1),^14^ resulting in the transition around *T*_2_ ≈ 24.15 K.^2,3,5^

To further investigate the magnetic transition near 26 K in CNO, the magnetocaloric effect (MCE) was analyzed through the magnetic entropy change, Δ*S*_M_, which is highly sensitive to subtle variations in magnetization, as a function of temperature under different applied magnetic fields, μ_0_ *H*. For this, magnetization isotherms were recorded in the temperature range of 12–36 K, with temperature intervals Δ*T* = 2 K and under magnetic fields up to 7 T (see the inset of Figure 5c). The curves flatten with increasing temperature and below *T*_N_, almost linear curves can be observed. The obtained data were subsequently used to calculate an Arrott-plot (*H*/*M *–* M* ^2^),^36^ depicted in Figure 5(c), which can be used to determine the order of the magnetic phase transition. Figure 5c clearly shows that the slopes for all curves are positive; therefore the magnetic ordering phenomenon can be classified as second-order magnetic phase transition (SOPT) in CNO. Finally, the magnetic entropy change Δ*S*_M_ was estimated using the following relation:

$$\Delta S_{M}\left(T,H_{0}\right)=\underset{i}{\sum }\frac{M_{i+1}-M_{i}}{T_{i+1}-T_{i}}{\Delta H}_{i}$$ 4

there by *M*_*i*_ and *M*_*i*+1_ denote the experimental data obtained at temperatures *T*_*i*_ and *T*_*i*+1_ under a magnetic field strength *H*_*i*_, respectively.^37^ Figure 5d shows the Δ*S*_M_(*T*) curves for CNO at various magnetic fields change ranging from 0 to 1 to 0–7 T. All curves exhibit a positive broad trace peak near the Neel temperature of *T*_1_ = 26 K, in which Δ*S*_M_^max^ (4.72 × 10^–3^ J/kg K at 26 K) value is progressively increasing with the μ_0_ *H*. Such behavior is typical of conventional MCE at magnetic phase transitions. The maximum magnetic entropy change, Δ*S*_M_^max^ ≈ μ_0_ *H* ^*n*^, was found to follow a power-law dependence on the applied magnetic field, as shown in the inset of Figure 5d. The extracted exponent, *n* = 1.73 ± 0.05, differs significantly from the ideal mean-field ferromagnetic value of *n* = 3/2.^38,39^ This deviation from the mean-field prediction indicates the possible presence of magnetic fluctuations in the vicinity of the transition temperature. In addition, the pronounced increase highlights the potential for enhancement of the MCE through variation of the applied magnetic field intensity.

### 3.5 EPR Spectroscopy

To probe the local electronic structure and magnetic interactions in CNO system, we performed the Electron Paramagnetic Resonance (EPR) spectroscopy, as this technique is particularly sensitive to paramagnetic Cu^2+^ centers (3d^9^, *S* = 1/2), enabling characterization of their oxidation state, coordination geometry, and spin–spin couplings. The EPR spectrum measured at 300 K for the CNO sample is shown in Figure 6. The peak-to-peak line width (Δ*H*_pp_) is defined as the peak-to-peak distance. The effective *g*-factor is experimentally defined by *g* = *h*ν/β*H*_r_, where ν is the microwave frequency, *H*_r_ is the resonance magnetic field at which the photon energy matches the separation between the electronic energy levels, *h* is Planck’s constant, and β is the Bohr magneton.^40^

**Figure 6:**
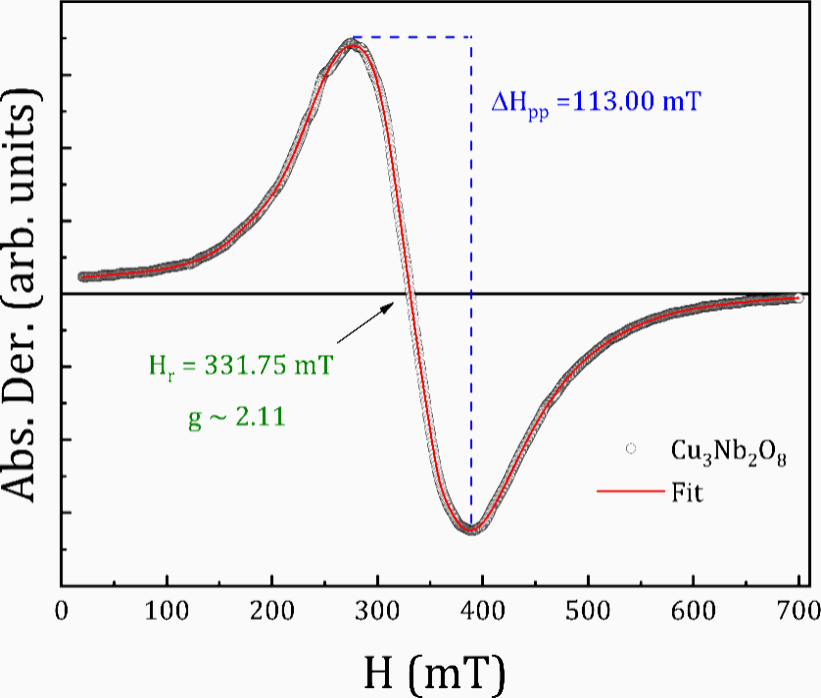
EPR spectrum for CNO sample measured at room temperature.

All values were extracted from the Gaussian fit of the experimental EPR data. The fitting gave values of Δ*H*_pp_ = 113.00 mT, *H*_r_ = 331.75 mT, and *g* = 2.11. Particularly, the observed *g*-value of 2.11 agrees well with reported values for superexchange-coupled Cu^2+^ pairs, confirming the presence of magnetic Cu^2+^ ions (*S* = 1/2) in the CNO structure. These ions mediate AFM superexchange interactions through Cu^2+^–O^2–^–Cu^2+^ pathways, as commonly observed in copper-oxide systems, which is also consistent with our observed magnetic behavior.

### 3.6 Evidence of Magnetoelastic Coupling in Cu_3_Nb_2_O_8_

The Rietveld refinement of the room-temperature SXRD data [Figure 1a] indicates that the compound crystallizes in the triclinic *P*1 space group, in agreement with the Raman spectroscopy analysis [Figure 4a] and previous reports.^2,3,5,14,16,17^ Nevertheless, earlier neutron diffraction studies identified a noncentrosymmetric magnetic phase belonging to the *P*1 space group below the magnetic ordering temperature.^3,5^ Herein, two transition temperatures are observed in the derivative of the susceptibility curve, shown in the inset of Figure 5b, which are consistent with the measurements of specific heat^2,3,5^ and magnetic susceptibility.^2^ As mentioned earlier, *T*_1_ = 26 K is attributed to a magnetic ordering transition, while *T*_2_ = 24 K is associated with the formation of a helical spin structure that magnetoelectrically couples to spontaneous polarization.^2,3,5^ Recently, Numan, M. et al.,^4^ revealed through temperature-dependent powder XRD and X-ray absorption fine structure (EXAFS), distinct anomalies in lattice parameters as well as considerable changes of Cu–O bond lengths in the first coordination sphere above *T*_N_. Below *T*_N_, the Cu(1)–O(1) and Cu(1)–O(2) bond lengths increase significantly, while the Cu(2)–O(1) and Cu(2)–O(2) bond lengths decrease with decreasing temperature.^4^ These variations in bond lengths indicate that the crystal lattice is flexible and strongly interconnected with the spin subsystem. Consequently, the sensitivity of vibrational spectroscopy can be exploited to correlate lattice dynamics with magnetic configurations in magnetically driven multiferroic systems. To gain deeper insight into the lattice distortions induced by magnetic ordering, as well as the spin–lattice interaction mechanism, the temperature-dependent Raman spectra of CNO were investigated. Figure 7a shows the Raman spectra recorded from 10 to 300 K. In general, the magnetoelastic coupling is evidenced by changes in the vibrational frequency at different magnetic ordering temperatures.^41^ The absence of additional peaks suggests that the crystal symmetry is preserved throughout the entire investigated temperature range, confirming that the magnetic anomalies are not associated with any structural phase transition.

**Figure 7:**
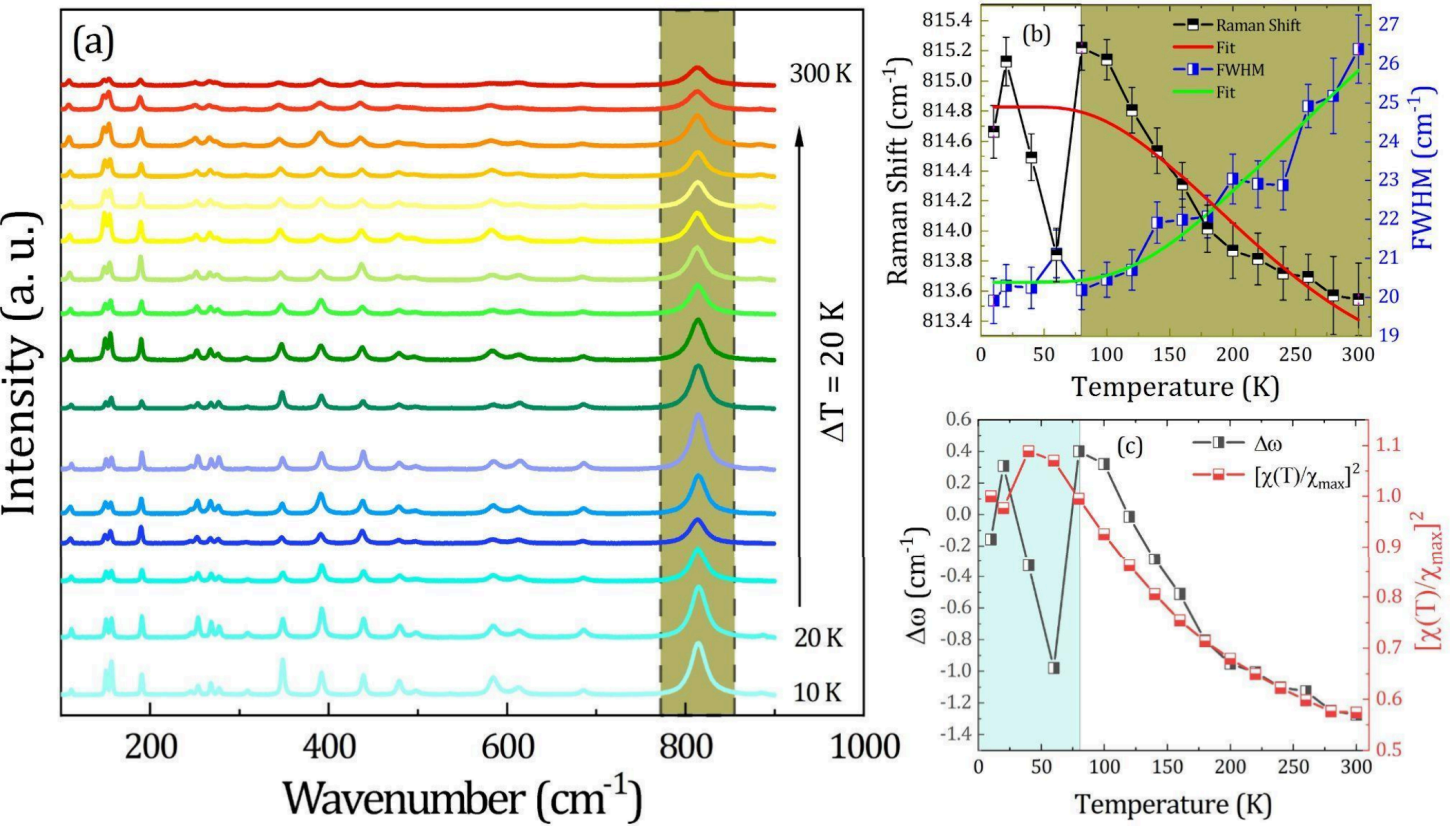
(a) Raman spectra of CNO as a function of temperature from 10 to 300 K, and (b) Raman shifts and FWHM as a function of temperature for modes localized at 814 cm^–1^. Fitting of experimental data to the Balkanski model, from eq 5 (red curve) to eq 6 (green curve). (c) Phonon shift (ω_obs_ *–* ω_0_) and squared normalized magnetic susceptibility ([(*T*)/χ_max_]^2^) as a function of temperature for the 814 cm^–1^ modes.

To further investigate the dependence of the phonon frequencies, we fitted the data considering the Balkanski model, which describes the anharmonic temperature dependence of the phonon modes. The temperature dependence of the phonon frequencies for the 814 cm^–1^ mode together with the fit assuming a standard anharmonic dependence of the phonon modes is shown in Figure 7b. We analyze Raman modes using a Lorentzian function. The experimental results were fitted using the Balkanski model, which describes the temperature dependence of the phonon wavenumbers by considering only anharmonic phonon contributions, as expressed by the following equation:^42–44^

$$\omega (T)=\omega_{0}+C_{1}\left(1+\frac{2}{e^{\hbar \omega_{0}/2k_{B}T}-1}\right)+C_{2}\left[1+\frac{3}{e^{\hbar \omega_{0}/3k_{B}T}-1}+\frac{3}{(e^{\hbar \omega_{0}/3k_{B}T}-1{)}^{2}}\right]$$ 5

as well as the dependence of the full width at half-maximum:

$$\Gamma (T)=\Gamma_{1}\left(1+\frac{2}{e^{\hbar \omega_{0}/2k_{B}T}-1}\right)+\Gamma_{2}\left[1+\frac{3}{e^{\hbar \omega_{0}/3k_{B}T}-1}+\frac{3}{(e^{\hbar \omega_{0}/3k_{B}T}-1{)}^{2}}\right]$$ 6

In this model, ω_0_ is the temperature-independent part of the frequency, *C*_1_, *C*_2_, Γ_1_ and Γ _2_ are adjustable parameters. The terms associated with *C*_1_ and Γ_1_ corresponds to contributions from three-phonon decay processes, whereas the terms associated with *C*_2_ and Γ_2_ account for four-phonon decay processes. In addition, *ℏ*ω_0_ denotes the phonon energy and *k*_B_ is the Boltzmann constant.^44^ The inclusion of higher-order anharmonic contributions is essential for an accurate description of the lattice dynamics, particularly in complex oxides where multiphonon scattering processes strongly affect the phonon self-energy.^45,46^ The parameters obtained from the fitting procedure are listed in Table S2.

Above 60 K, the Raman modes show a gradual shift to low frequency as the temperature increases, following the behavior described by the fitted model (eqs 5 and 6), accompanied by a monotonic increase in fwhm, Figures 7b and S5. This is mainly due to the thermal expansion of the lattice as the thermal energy increases.^43^ Below 60 K, the experimental data diverge notably from the Balkanski fit, revealing anomalies in both Raman shift and fwhm (Figure 7b). To quantitatively evaluate this behavior, we determined the frequency deviation defined as δω_s‑ph_ = ω_0_ *–* ω_anh_(*T*), where ω_anh_ represents the frequency calculated from the Balkanski model. The noticeable “dip” observed around 60 K corresponds to the maximum value of δω_s‑ph_, providing a quantitative measure of the nonanharmonic contributions, Figure 7c. Such deviations from purely anharmonic models indicate that the lattice dynamics are being modulated by nonthermal contributions, as observed in other multiferroic systems.^47,48^ Notably, such anomalous phonon shifts, occurring at temperatures much higher than the magnetic ordering transition temperature, are similar behavior is reported for other multiferroics,^41,49^ although in this case the deviation appears to be a spin-phonon coupling effect.^41,50^ This coupling can be modeled by the relation:

$${\delta \omega }_{s-\mathrm{ph}}(T)=\lambda \langle S_{i}\cdot S_{j}\rangle \approx [\chi (T)/\chi_{\max}{]}^{2}$$ 7

where λ is the spin-phonon coupling constant and ⟨*S*_*i*_ *·S*_*j*_⟩ is the spin–spin correlation function, and χ­(*T*) is the magnetic susceptibility.^51^ Figure 7c shows the phonon shift (δω) and squared normalized magnetic susceptibility ([χ­(*T*)/χ_max_]^2^) data as a function of temperature, where we can observe that there is good agreement with eq 7. From this deviation, we calculated the spin-phonon coupling factor, λ, for the 814 cm^–1^ mode as −5.56 cm^–1^, respectively. Both modes exhibit clear anomalies between *T* = 20–60 K (kinks in ω­(*T*) and Γ­(*T*), extreme in δω_s‑ph_(*T*), consistent with AFM spin–phonon coupling. Since the anomalies emerge close to *T*_1_, it is reasonable to assume that short-range spin correlations begin to develop within this temperature region. Such correlations originate from magnetic frustration are due to frustration and become more pronounced as they approach the magnetic ordering transition series.^41^ The softening or hardening of the approach mode demonstrates the sensitivity of the lattice to the magnetic state via exchange-striction mechanisms. Clearly, the observed frequency shifts reflect both local structural distortions within the lattice and variations in the spin–phonon coupling strength.^41^ Thus, we can associate the magnetic anomalies observed in the Raman spectroscopy data with contributions from magnetic (spin-phonon) and lattice (spin–lattice) order.

## 4 Conclusion

In summary, we have studied the structural, vibrational, electronic, and magnetic properties of the copper orthoniobate Cu_3_Nb_2_O_8_. SXRD analysis confirmed the triclinic *P*1̅ symmetry, while Raman and infrared spectroscopy revealed all symmetry-allowed vibrational modes, supported by lattice dynamics calculations that accurately reproduce both phonon wavenumbers and atomic displacement patterns. DFT calculations elucidate the electronic structure, demonstrating that while the conduction band minimum derives primarily from Cu 3d orbitals, the valence band is dominated by O 2p states with negligible contributions from transition metal cations near the Fermi level. Magnetic measurements have revealed two distinct AFM transitions at *T*_1_ = 26.09 K and *T*_2_ = 24.15 K, in which the spin-phonon coupling from temperature-dependent Raman spectra (20–60 K) analysis provided direct evidence of magneto-structural correlations. The MCE and second-order phase transition (SOPT) observed near *T*_N_ suggest the presence of magnetic fluctuations mediated by superexchange interactions through Cu^2+^–O^2–^–Cu^2+^ pathways. These findings regarding the coupling of the lattice, electronic, and spin degrees of freedom, as well as the multiple low-temperature transitions, make Cu_3_Nb_2_O_8_ an intriguing material for studying correlated phenomena in mixed 3d/4d transition metal oxides.

## Declaration of AI-Assisted Technologies in the Preparation of the Manuscript

During the preparation of this manuscript, the authors used artificial intelligence tools to support language improvement, formatting, and table organization. All generated content was carefully reviewed and revised by the authors, who assume full responsibility for the final version of the manuscript.

## Supplementary Material



## Notes

X-ray diffraction in powder; table with refinement data; partial density of states; polarized absorption; magnetic susceptibility adjustment; Raman shift and full-width at half-height (FWHM) as a function of temperature; fitting of the inverse susceptibility curve to the modified Curie-Weisss law; rietveld refinement data, atomic positions, lattice parameters, quality factors, bond angles and lengths; and parameters extracted from the Balkanski model adjustment (PDF)
