# Supplementary material for: Magneto-Structural Coupling in the Antiferromagnetic Copper Orthoniobate Cu3Nb2O8
Source: ACS Omega. 2026 Jun 23;11(26):38945–57. doi: 10.1021/acsomega.6c02538 (PMC13347367; doi:10.1021/acsomega.6c02538)
Supplement: Supplementary file 1 [file ao6c02538_si_001.pdf]

Supplementary material

Magneto-Structural Coupling in the Antiferromagnetic Copper Orthoniobate $Cu_3Nb_2O_8$

Magneto-Structural Coupling in the Antiferromagnetic Copper Orthoniobate $Cu_3Nb_2O_8$

Diego da Silva Evaristo[1], Romualdo Santos Silva Jr.[2], Raí Figueredo Jucá[3], Luiz Fernando Lobato da Silva[4], Gilberto Dantas Saraiva[1], Waldeci Paraguassu Feio[5], Javier Gainza[6], João Elias Rodrigues[6], José Luis Martínez[2], José Antonio Alonso[2], Nilson dos Santos Ferreira[3], Marcelo Andrade Macêdo[3] and Antônio Joel Ramiro de Castro*[7]

[1]Faculty of Education Sciences and Letters of Sertão Central, State University of Ceará, Quixadá 63902-098, Brazil.

[2]Instituto de Ciencia de Materiales de Madrid (ICMM), CSIC, E-28049 Madrid, Spain.

[3]Department of Physics, Federal University of Sergipe, São Cristóvão, 49100-000, Sergipe, Brazil.

[4]Institute of Criminalistics, Scientific Police of Pará, Marabá, PA, 68507-000, Brazil.

[5]Institute of Exact and Natural Sciences, Federal University of Pará, 66075-110, Belém do Pará, Brazil.

[6]European Synchrotron Radiation Facility (ESRF), 71 Avenue des Martyrs, 38000 Grenoble, France.

[7]University Federal of Ceará, 63902-580, Quixadá, CE, Brazil.

* **Corresponding author:** Antonio Joel Ramiro Castro, **E-mail address:** joelcastro@fisica.ufc.br

Data analysis-Rietveld refinement

The structural characterization was analyzed by the X-ray diffraction (Rigaku) using Cu-Kα radiation (λ = 1.54 Å) in the range of 10-90°, the step of 0.02° and counting time by step of 2s. The crystal structures lattice parameters were refined using the Rietveld method with the GSAS I Software.[1]

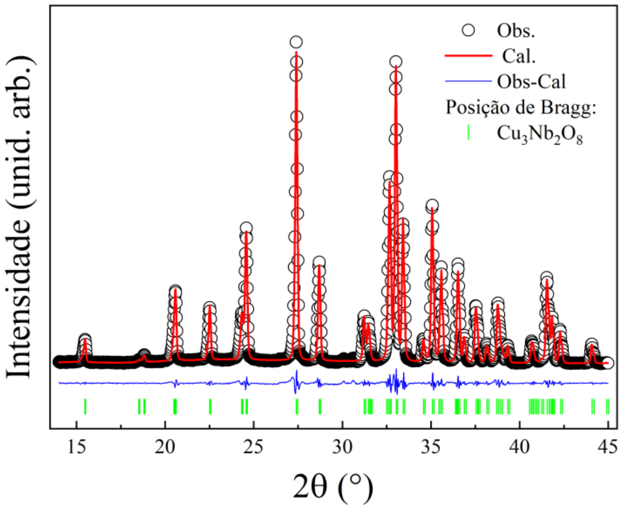

Fig. S1. Rietveld refinement of the X-ray diffraction pattern for $Cu_3Nb_2O_8$.

Table S1. Rietveld refinement data, atomic positions, lattice parameters, quality factors, bond angles and lengths.

Atom	*Pos. Wyck.*	*X*	*y*	*y*	*Occup.*	*B (10^{-3})*
Cu1	1a	0.0000(0)	0.0000(0)	0.0000(0)	0.98(4)	5.09(1)
Cu2	2i	0.4771(8)	0.0527(9)	0.2372(9)	0.99(3)	1.20(2)

Nb	2i	0.2160(5)	0.5388(6)	0.6541(9)	0.98(5)	0.77(6)
O1	2i	0.2291(4)	0.1509(9)	0.9392(5)	1.00(0)	2.84(3)
O2	2i	0.2718(4)	0.7346(0)	0.8271(0)	1.00(0)	1.67(1)
O3	2i	0.3249(4)	0.7439(4)	0.3691(5)	1.00(0)	4.68(7)
O4	2i	0.1853(7)	0.2792(1)	0.4111(0)	1.00(0)	0.12(8)
Lattice parameters:						
a = 5.1720(7) (Å)		b = 5.4714(0) (Å)		c = 6.0002(4) (Å)		V = 147.5(7) (Å^3)
α = 72.528(7) (°)		β = 83.415(2) (°)		γ = 65.672(4) (°)		ρ = 5.520 (g/cm^3)
Quality factors: χ^2 = 12.28, RF = 1.17 (4) %, W_{RP} = 2.13%, RP = 1.51%						
bond length (Å) and angle [°]:						
Cu(1)-O(1)	Cu(1)-O(2)	Cu(2)-O(1)	Cu(2)-O(1)	Cu(2)-O(2)	Cu(2)-O(3)	Cu(2)-O(4)
1.6573(0)	2.0123(7)	1.9019(0)	2.1245(8)	2.0116(5)	2.0620(7)	1.9428(2)
Cu(1)-O(1)-Cu(2)	111.503(6)	Cu(1)-O(2)-Cu(2)	90.330(3)			

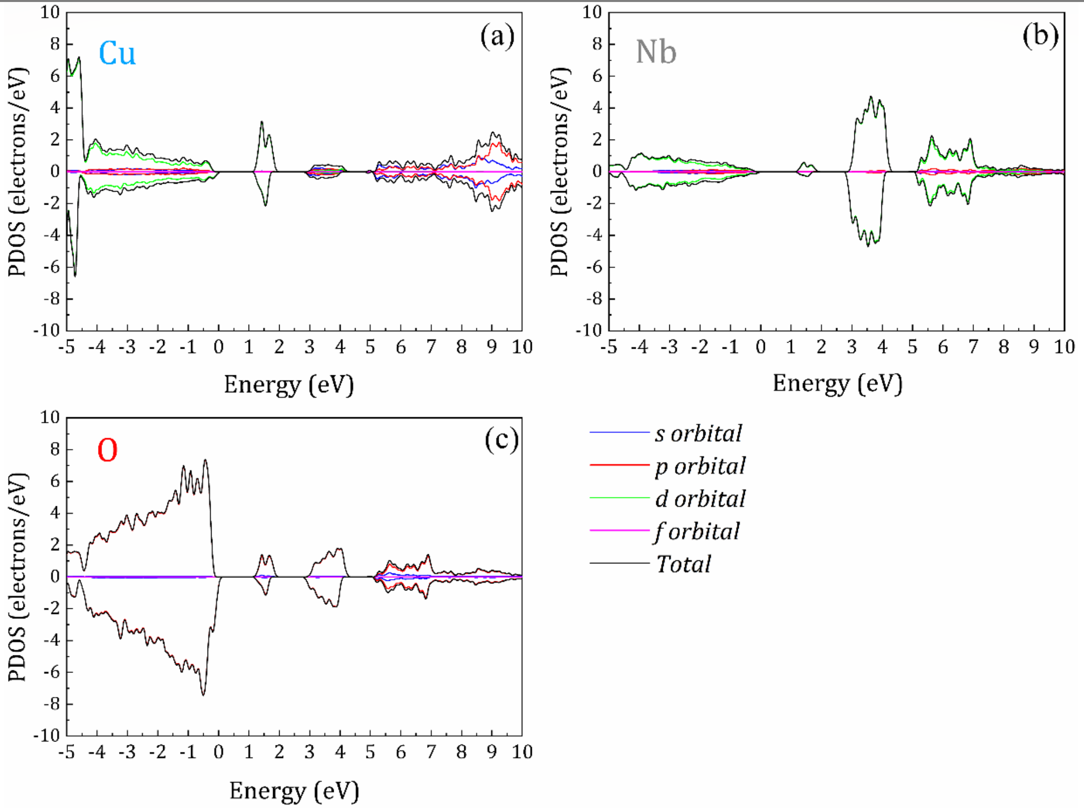

Fig. S2. Partial Density of States (PDOS) selected by atom contribution: (a) Cu, (b) Nb and (c) O.

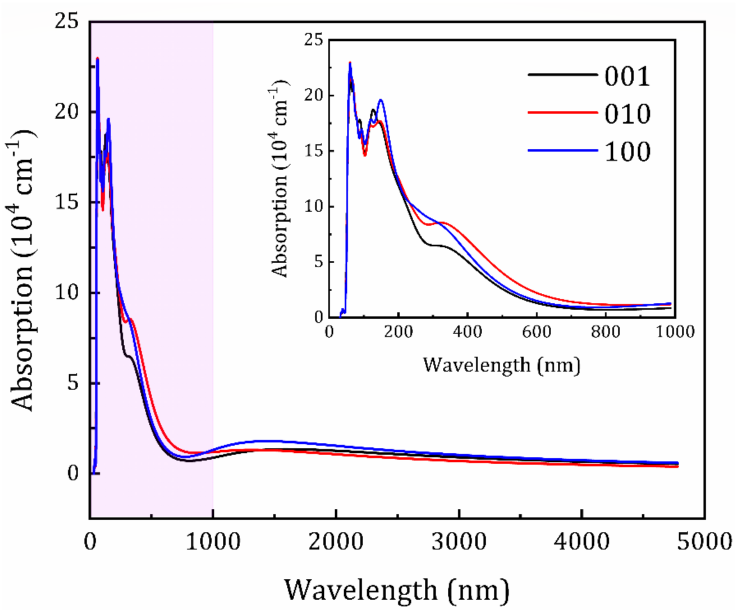

Fig. S3. Polarized absorption for the $Cu_3Nb_2O_8$ structure.

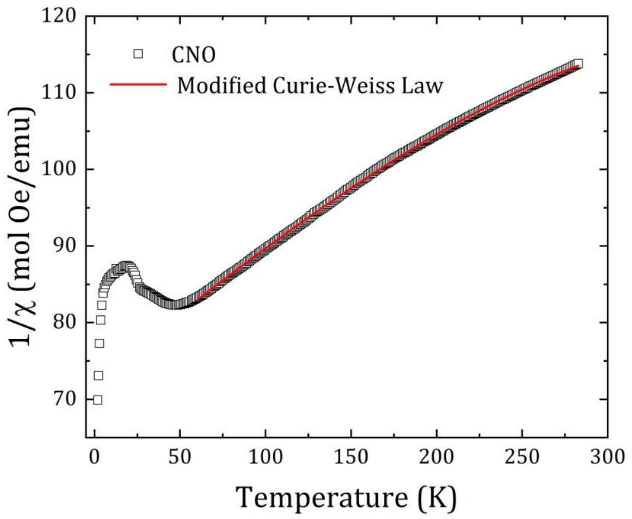

Fig. S4. Fitting of the inverse susceptibility curve to the modified Curie-Weisss law for the $Cu_3Nb_2O_8$ measured in an external field H = 100 Oe.

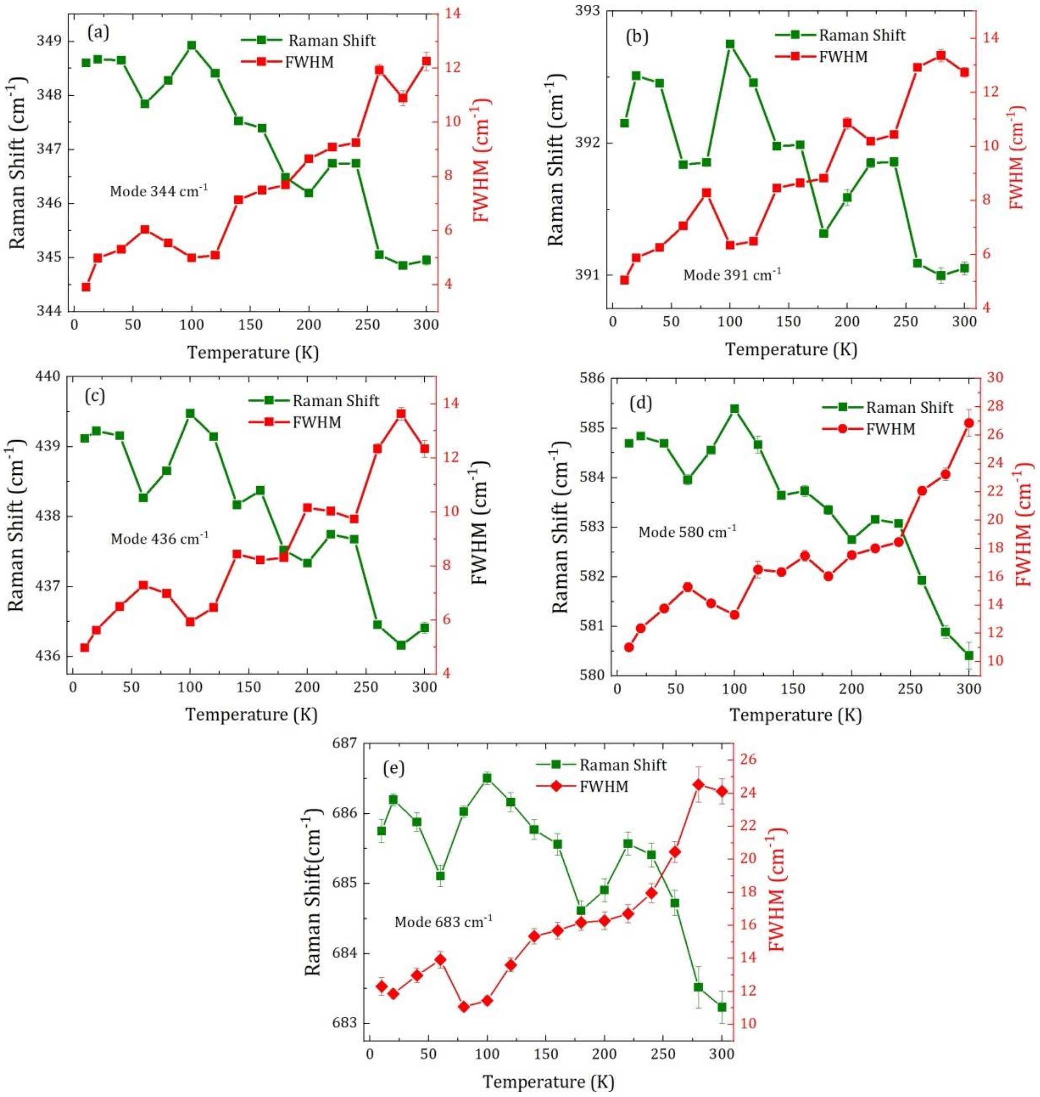

Fig. S5. Raman shift and FWHM as a function of temperature for the modes: (a) 344 cm^{-1}, (b) 391 cm^{-1}, (c) 436 cm^{-1} (d) 580 cm^{-1}, and (e) 683 cm^{-1}.

Table S2: Parameters extracted from the Balkanski model adjustment, Eq. (5) and (6).

**Parameters**	**Mode 814 cm^{-1}**
**ω_0 (cm^{-1})**	818.63 $\pm$ 1.82
**C_1 (cm^{-1})**	-4.12 $\pm$ 3.33
**C_2 (cm^{-1})**	1.05 $\pm$ 1.28
**Γ_1(cm^{-1})**	29.75 $\pm$ 3.29
**Γ_2 (cm^{-1})**	-9.36 $\pm$ 3.35

References

[1] B. H. Toby, EXPGUI, a graphical user interface for GSAS, *Journal of Applied Crystallography* **2001**, 34, 210-213.
